# Membrane fouling monitoring by 3ω sensing

**DOI:** 10.1038/s41598-023-42337-1

**Published:** 2023-09-14

**Authors:** Mads Koustrup Jørgensen, Frederikke Kildeberg Paulsen, Anders Bentien, Astrid Ræbild Kjul, Maiken Poulsen, Louise Mailund Mikkelsen, Nikitha Thavaneswaran, Simon Abildgaard Hansen, Pernille Krogsager Jensen, Jacob Andersen, David N. Østedgaard-Munck, Jan Dimon Bendtsen, Morten Lykkegaard Christensen

**Affiliations:** 1 Center for Membrane Technology, Department of Chemistry and Bioscience, Aalborg University, 9220 Aalborg East, Denmark; 2 Department of Biological and Chemical Engineering, Aarhus University, Åbogade 40, 8200 Aarhus N, Denmark; 3 LiqTech International A/S, Benshøj Industrivej 24, 9500 Hobro, Denmark; 4 Department of Electronic Systems, Aalborg University, Fredrik Bajers Vej 7, 9220 Aalborg East, Denmark

**Keywords:** Chemical engineering, Process chemistry, Characterization and analytical techniques

## Abstract

Membrane fouling significantly reduces membrane permeability, leading to higher operational expenses. In situ monitoring of membrane fouling can potentially be used to reduce operation cost by optimizing operational parameters and cleaning conditions. In this study, a platinum wire with a diameter of 20 µm was attached to the surface of a ceramic ultrafiltration membrane, and by measuring the voltage across the wire while applying an AC current, the amplitude of the third harmonic wave, the so-called 3ω signal, was obtained. Results showed increasing 3ω signals during formation of fouling layers, which correlates directly to the hydraulic resistance of the formed fouling layer in semi-dead end filtration of polymeric core shell particles and crossflow filtration of diluted milk. This is explained by the insulating effect of the fouling layers which reduces heat convection by crossflow and the different thermal conductivity in the fouling layer compared with the feed. After membrane cleaning, the permeability and the magnitude of the 3ω signal were partly restored, showing that the 3ω method can be used to monitor the effect of cleaning. The frequency of the AC current was varied so it was possible to measure the heat conductivity in the fouling layer (high frequency) and heat convection due to cross-flow (low frequency). This may potentially be used to get information of the type of fouling (heat conductivity) and thickness of the fouling layer (AC frequency where heat conductivity becomes dominating).

## Introduction

Membrane filtration is an efficient and continually evolving technology for separation and purification in water treatment, wastewater treatment, and in the food and pharmaceutical industries. However, membrane performance is limited by fouling, where material on and within the membrane surface is accumulated^1^. Fouling covers a range of mechanisms, such as cake and biofilm formation (external, to some extent reversible), scaling, adsorption and pore blocking (internal and often irreversible). Fouling reduces performance in terms of higher operational expenses, lower capacity and changes in selectivity^1,2^. For reverse osmosis, fouling has been assessed to account for about 25% of the operational expenses^3^, whereas for membrane bioreactors for wastewater treatment, costs associated with fouling account for 30–70% of operational costs^4^. Fouling is inevitable, and the best strategy is to control rather than avoid fouling. This calls for efficient monitoring of fouling locally and in real time for rapid responses to reduce fouling by, for example, physical cleaning, reducing flux or elevating crossflow^1,5^. Currently, membrane fouling monitoring in full scale is mainly monitored by transmembrane pressure measurement (TMP) and flux measurements^1,6^. The low spatial and temporal resolution of these fail to show local onset of fouling before it spreads, and often the transmission of compounds to be recovered (e.g. proteins) is reduced before TMP or flux measurements can reveal fouling^1,7^. Fouling is mitigated by regular cleaning procedures, nonetheless the offset conditions for a cleaned membrane surface are based on trial and error as the degree of cleaning is not monitored during cleaning. Clearly, real-time monitering of the degree of the cleaning has a large potential in terms of the selection of cleaning agent and cleaning time (downtime) in order to reduce costs associated with cleaning and prolong and membrane lifetime^1^.

For direct measurement of the state of the membrane, different technologies have been proposed for fouling monitoring, including ultrasonic reflectometry^8,9^, laser triangulometry^10^, direct observation through microscope^11,12^, Brunauer-Emmet-Teller adsorption analysis^13^, and Raman spectroscopy^7,14–16^. However, these methods have low resolution or require optical windows (i.e., non-turbid suspensions) and are mainly limited to lab scale. Electrical impedance spectroscopy (EIS)^7,15,16^ can also measure the fouling formation by measuring the change in capacitance, giving a high resolution of measurement. However, the method is limited to early-stage fouling monitoring as an upper threshold limit is observed for organic fouling. Another electrically based method for fouling monitoring is streaming potential, which measures the change in surface charge as a membrane is fouled.

A promising alternative method for fouling monitoring by collection of electrical signals is by the measurement of change in heat transfer near membranes as it is non-invasive and does not require an optical window. It has also been shown that biofilm formation can be measured by a heat transfer sensor^17^. In membrane filtration, heat transfer has been measured by constant temperature anenometry to monitor flow distribution on membrane surfaces^18,19^. This method applies a heating current to a thin platinum wire that is integrated into the feed side spacer of a membrane. The wire voltage is measured and used to determine the convective heat transfer from the heated element to the surrounding fluid, which has been shown to depend on fluid velocity^18^. The method can describe the heterogeneity of fouling across a membrane, and detect areas with poor aeration or crossflow^19^.

A more advanced technique for measurement of thermal conductivity is the 3ω method^20^. It is an established technique to measure thermal conductivity of solid samples, but has not yet been applied for membrane fouling measurement. In the 3ω method, the same wire serves the dual purpose of generating heat and measuring thermal conductivity, which enables more precise measurements of the fouling layer at the exact location where membrane fouling occurs. Over the past decade, the 3ω method has been used for the measurement of, among other things, flow speeds^21^ and cell viability^22^. It has been shown that the 3ω signal can be used to measure layer deposits around a sensor surface, however, not yet for membrane applications but only for non-permeable surfaces^23^. An AC current with angular frequency (ω) was passed through a thin metal film deposited on a substrate and, due to Joule heating, the temperature of the thin film and the surroundings oscillated with a frequency twice that of the base frequency (2ω). As a result of the temperature coefficient of resistance for the metal film, the resistance of the metal film also oscillates with 2ω. The measured electrical potential difference across the film will, due to Joule heating, include a complex signal at a frequency three times the base frequency (3ω). The amplitude of this voltage (*U*_3ω_) is determined by the heat conduction surrounding the sensor; in particular, *U*_3ω_ increases if a thermally insulating material deposits on the sensor surface, as the thermal conductivity of the foulant is different from the thermal conductivity of the suspension surrounding the sensor. For example, fat and casein have been reported to have thermal conductivities of 0.21 W m^−1^ °C^−1^ and 0.20 W m^−1^ °C^−1^, respectively, which are lower than the thermal conductivity of water at 0.598 W m^−1^ °C^−1^^24^. In contrast, compounds with a higher thermal conductivity than water, CaCO_3_ (2.7 W m^−1^ °C^−1^), for example, will lead to a reduction in *U*_3ω_. In addition, a higher amount of deposit will lead to a larger change in signal amplitude^23^. Thus, the signal depends on thermal conductivity and amount of deposit, which has been demonstrated with a sensor on the surface of a plate showing higher 3ω signal at greater deposit thicknesses and lower 3ω signal after cleaning^23^. It was also shown that the convection of heat by a water flow across or along the circuit affects the 3ω signal^23^. Based on this result, it is hypothesized that for membrane filtration a high water velocity through (permeation) or along (crossflow) the membrane will lead to a reduction in the 3ω signal, which will enable the flow conditions near a membrane and through the membrane to be described. Another feature of the method is that the penetration depth of thermal conductivity is frequency dependent^23^. The thermal penetration depth (thermal wavelength) is the depth that heat diffuses when heated in one AC cycle and therefore a rough measure of the maximum depth of measuring thermal conductivity^20,23^. When AC frequency is reduced, the thermal penetration depth increases, which affects the 3ω signal. Hence, by varying AC frequencies on the sensor below a fouling layer of a given thickness, constant 3ω signals can be expected for penetration depths smaller than the fouling layer thickness, as the signal will only depend on fouling layer thermal conductivity and not the amount of fouling. This feature may enable characterization of different fouling types (organic/inorganic) or fouling layer compactness; specifically, it may distinguish between foulant layers with different thermal conductivities. Further, AC frequency can be reduced to reach a penetration depth greater than the fouling layer thickness, allowing the 3ω signal to measure fouling thickness. The potential dual information of monitoring amount and composition or characteristics of fouling layers makes the 3ω method novel compared to existing methods.

This study will investigate the 3ω method for measuring thermal conductivity of membranes, monitor flow conditions, and the formation of fouling layers in real time in membrane filtration. A 20 µm thick platinum wire is attached to the surface of a ceramic UF membrane, and the 3ω signal is measured under varying conditions, to assess the ability of the method to measure changes in flow velocity along the membrane, presence of deposit on the membrane in varying amounts, and the formation of fouling layers by core–shell particles and milk in semi-dead-end and crossflow modes.

## Results

### Sensing environment around membrane

**Figure 1:**
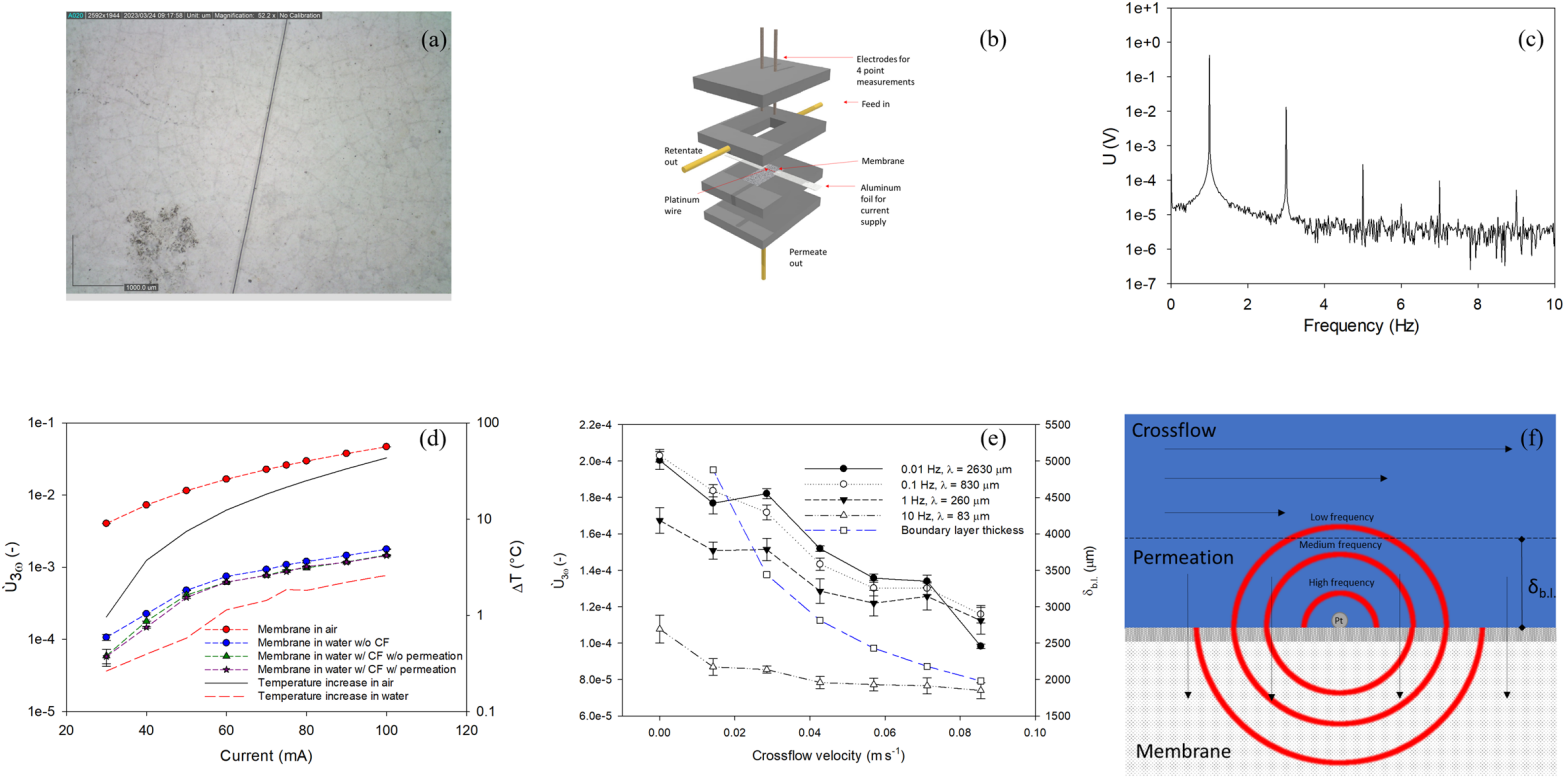
Microscopy image of platinum wire on ceramic membrane consisting of a selective ZrO_2_ layer with a SiC support, captured with Dino-Lite microscope (**a**) and the membrane filtration cell with platinum wire (Ø = 20 µm) integrated on the membrane (**b**). The Fourier transform of the measured voltage shows amplitudes at 1ω and 3ω; i.e., the U_3ω_ is determined from the amplitude at a frequency of 3 × ω (**c**). Ù_3ω_ signals measured at 1 Hz AC at varying currents on a platinum wire on a membrane in air and in water with varying stagnant, crossflow and permeation conditions (**d**). Ù_3ω_ signals were determined at 75 mA AC at different AC frequencies and varying crossflow velocities (**e**). (**f**) shows an illustration of a cross-section of the membrane with the thermal waves from the heated platinum wire on the ceramic membrane, and how the heat is released to the surrounding feed suspension, consisting of a laminar boundary layer and a crossflow beyond this, and membrane. Ù_3ω_ are averages with standard deviations of three replicates.

A 20 µm thick platinum wire was attached to a ceramic membrane installed in a membrane module for semi-dead end and crossflow filtration (Figs. 1a,b, S1) and *U*_3ω_ was measured on the wire at varying currents (30–100 mA) at a 1 Hz AC frequency. Measured voltages were treated by Fourier analysis to determine *U*_3ω_ and *U*_1ω_, the amplitude of the first harmonic wave (Fig. 1c). Signals were measured on wire on membrane in air, in DI water with and without crossflow (2.85 × 10^–2^ m s^−1^), and in DI water with crossflow and permeation (40 L m^−2^ h^−1^ permeate flux, corresponding to 1.11 × 10^–5^ m s^−1^), using a crossflow setup (Fig. S2a). The *U*_3ω_ signals were normalized with the voltage over the wire to give the normalized, temperature-independent signal (*Ù*_3ω_) in the manner proposed by Clausen et al.^23^ (Fig. S3).

*Ù*_3ω_ increases with current, which can be explained by Joule heating of the wire. Figure 1d summarizes the increasing *Ù*_3ω_ signals under the different conditions, along with the elevation in temperature of a platinum wire on a membrane in air and on a membrane in water. For the wire on a membrane in air, the estimated temperature increase from room temperature (*ΔR/R* ~ *ΔT/T*) increased from *ΔT* = 1.0 °C at 30 mA to *ΔT* = 43.3 °C at 100 mA. A comparison of the 3ω signals shows that *Ù*_3ω_ is reduced by the water, which has a higher thermal conductivity than air (thermal conductivities for water and air are 0.598 and 0.034 W m^−1^ °C^−1^, respectively). The calculated temperature change was also lower in water, increasing from *ΔT* = 0.3 °C at 30 mA to *ΔT* = 2.6 °C at 100 mA in water. The lower temperature change of wire in water is a result of the higher thermal conductivity and heat capacity of water than air. The low temperature increase in water does not affect any fouling layers deposited, which is essential for the 3ω method to act as a fouling sensor during filtration. The application of a crossflow resulted in a further reduction of *Ù*_3ω_, owing to enhanced heat convection. However, there was no significant reduction of *Ú*_3ω_ by permeation, which may be a result of the permeation velocity (1.11 × 10^–5^ m s^−1^) being orders of magnitude lower than the crossflow velocity (2.85 × 10^–2^ m s^−1^) and not significantly enhancing conduction of heat from the wire. This is confirmed by measurements of *Ù*_3ω_ at varying permeation fluxes (0.86 × 10^–5^–3.3 × 10^–5^ m s^−1^), which showed no effect on the signal (Fig. S4). To investigate the influence of crossflow further, *Ù*_3ω_ was measured at different crossflow velocities without permeation and with varying AC frequencies to vary penetration depth. There was a clear tendency toward the reduction of *Ù*_3ω_ at higher crossflow velocities (Fig. 1e). This result suggests that *Ù*_3ω_ is affected by changes in crossflow velocity across the heated wire, owing to the higher heat convection with increased crossflow, in accordance with previous studies^23^. The *Ù*_3ω_ magnitude decreases, expected from Eq. (3) (“Methods”), from low frequencies (0.01 Hz) to higher frequencies (10 Hz). However, the trend toward the reduction of *Ù*_3ω_ at higher crossflow velocities was less significant at the highest applied AC frequencies of 10 Hz. This result can be explained by the lower penetration depth at higher AC frequency, as described by Eq. (5) in “Methods”; that is, the measurement may be within the stagnant boundary layer, as illustrated in Fig. 1f. Measuring *Ù*_3ω_ at low frequencies with penetration depths towards the boundary layer thickness leads to the lowest values of *Ù*_3ω_ because of heat convection by the high crossflow. For lower AC frequencies, the penetration depth is smaller relative to the thickness of the boundary layer, but the measurement is still affected by enhanced heat convection. It should be noted that the applied crossflow velocity is lower than industrially applied crossflow velocities, which often are above 1 m s^−1^. Hence, a larger contrast between a fouled and non-fouled sensor can be expected, because of the higher thermal convection. The 20 µm thick wire mounted on the membrane may cause some disturbance and turbulence in the flow and boundary layer thickness. Future studies should investigate how the wire can be integrated within the membrane to avoid disturbing flow and fouling formation while also investigating flow at higher and more industrially relevant crossflow velocities.

### Sensing artificial fouling layers at varying AC frequencies

**Figure 2:**
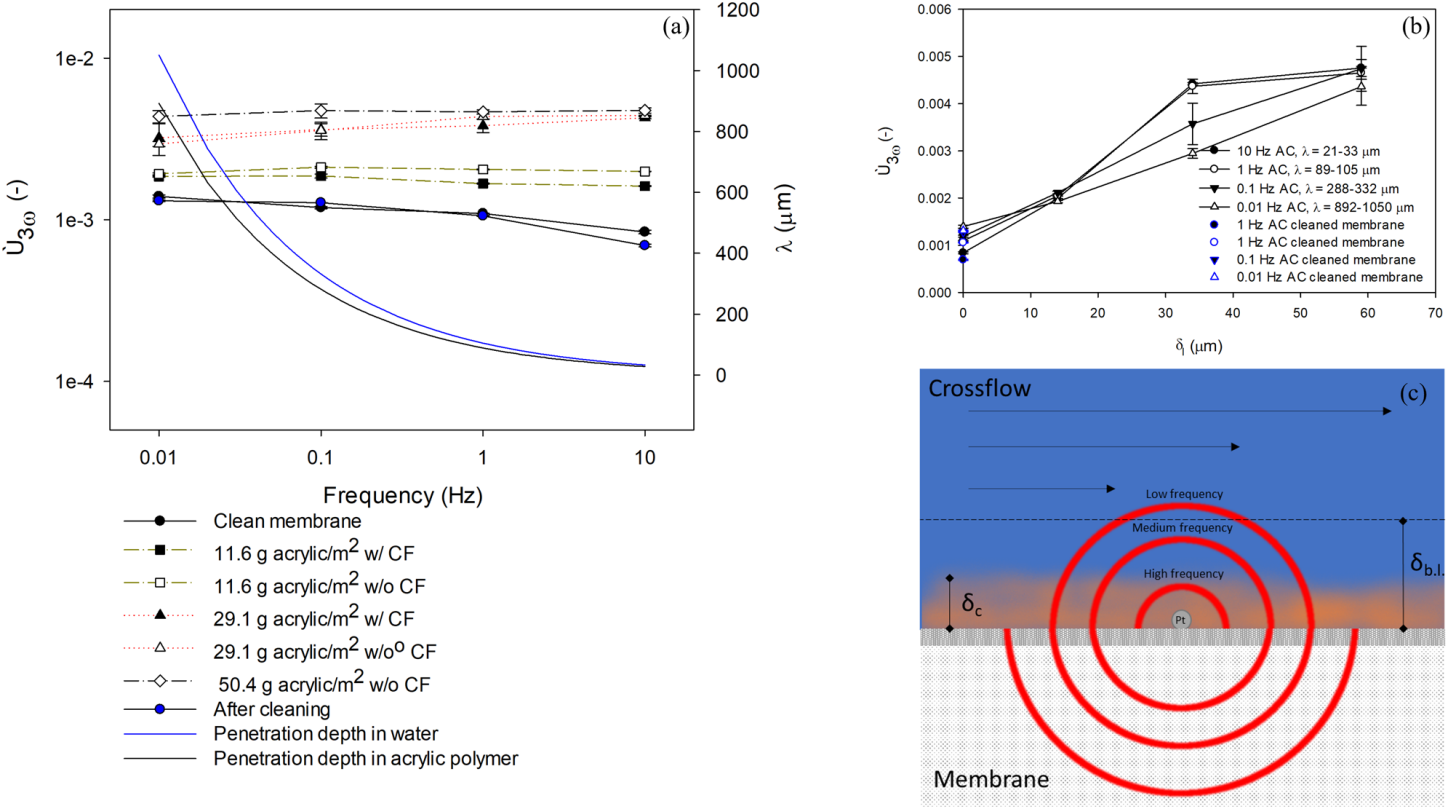
Ù_3ω_ signals (average values with standard deviations of three replicates) at varying frequencies obtained by AC of 75 mA through platinum on a clean membrane with crossflow of water and without permeation, a membrane with one layer of acrylic (11.6 g m^−2^) w/ and w/o crossflow, a membrane with two layers of acrylic (29.1 g m^−2^) w/ and w/o crossflow, a membrane with three layers of acrylic (50.4 g m^−2^) w/ crossflow and after removal of acrylic layers by acetone cleaning (**a**). The theoretical thermal penetration depths of water and acrylic polymer are plotted as a function of AC frequency, resembling a likely range of penetration depth in the fouling layer. Ù_3ω_ signals are plotted against estimated thicknesses of acrylic varnish on the membranes, showing an increasing signal with layer thickness for the applied AC frequencies (**b**). The illustration (**c**) shows how heat is released to the fouling layer and feed suspension from the wire at varying AC frequencies.

From the preceding experiments it was determined that an AC current of 75 mA was the best trade-off between a high *Ú*_3ω_ signal, while keeping the average temperature increase (*ΔT*) at a reasonable level. The membrane with a wire affixed to it was in turn covered in one, two, and three layers of acrylic varnish. For each layer, *Ú*_3ω_ was measured in stagnant DI water and DI water creating a crossflow velocity of 2.85 × 10^–2^ m s^−1^ in the crossflow setup. The mass of deposited varnish was found by comparing the dry weight of the membrane before and after coating. After analysis, the varnish was removed with acetone, and the clean membrane signal was measured in stagnant water. For comparison, signals were collected from the platinum wire on a clean membrane before the varnish covering. The resulting *Ú*_3ω_ signals are presented in Fig. 2a along with the estimated penetration depths in water and acrylic polymer.

The *Ù*_3ω_ signal measured on a membrane in water before application of varnish decreased with AC frequency, as expected. As a layer of acrylic varnish was applied, which by gravimetric analysis was found to cover 11.6 g acrylic m^−2^ membrane, *Ù*_3ω_ increased significantly by a factor of 1.4–2.4 compared to the clean membrane, depending on AC frequency. The signal was lower for a crossflow of water along the membrane compared to stagnant water above the membrane (Fig. 2a), as discussed in the previous section. As a second and third layer of acrylic was applied, gravimetric analysis showed that the membrane was covered by 29.1 g and 50.4 g acrylic m^−2^ membranes, respectively. *Ù*_3ω_ increased further depending on AC frequency. Here, no decline in signal with AC frequency was observed; instead, the signal was found to increase both with and without crossflow. This result can be seen in Fig. 2b, with *Ù*_3ω_ increasing with increasing layer thickness, as estimated by gravimetric analysis. However, for higher AC frequency measurements, the signal did not increase between layers with estimated thicknesses of 34–59 µm. This result is explained by the lower penetration depth in water and in acrylic polymer at higher AC frequencies, as depicted in Fig. 2c. As the penetration depth decreases, the *Ù*_3ω_ signal will to a greater extent reflect the lower thermal conductivity of the acrylic compared to water, with the *Ù*_3ω_ signal increasing. This result suggests that the AC frequency can be varied to study fouling in different ways, as illustrated in Fig. 2c, and will be subject for further studies. At high AC frequencies and a low penetration depth (less than the fouling layer thickness), the *Ù*_3ω_ signal will reflect the thermal conductivity of the fouling layer. Consequently, it may be possible to use the method to distinguish between different types of fouling with different thermal conductivities (for example, scaling vs. organic fouling). Reducing the AC frequency and increasing the penetration depth results in the penetration depth becoming larger than the fouling layer. The *Ú*_3ω_ signal depends on both the thickness of the fouling layer and heat convection. Furthermore, the fouling layer insulates against or promotes heat transfer depending on thermal conductivity. Therefore, the method may serve as a tool to distinguish changes in fouling, differentiating between amount of fouling and fouling material simply by measuring the *Ú*_3ω_ signal at varying AC frequencies with penetration depths reaching within and outside the fouling layer. The ability to distinguish between amount and type of membrane fouling is a unique feature of this technology, which has not been reported elsewhere^1^. This ability to discriminate between growth of fouling layers and changes in the nature (compactness, composition) from local, real-time measurements of fouling may potentially serve as a valuable tool for the choice of cleaning strategy in membrane module design and operations.

### Sensing fouling during semi-dead-end filtration

**Figure 3:**
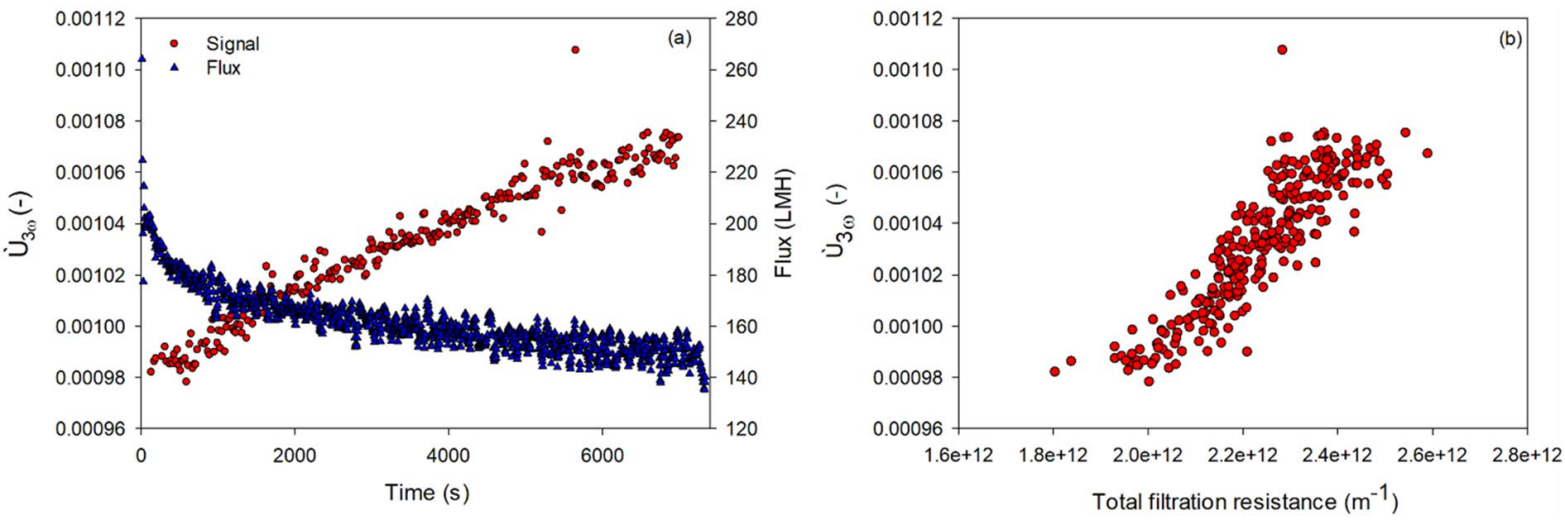
Development in flux and Ù_3ω_ measured at 75 mA AC with 1 Hz frequency over time during semi-dead-end filtration of PS-PAA particles at 1 bar (**a**) and Ù_3ω_ plotted against total hydraulic resistance to filtration (**b**).

The ability to sense fouling was tested by *Ù*_3ω_ measurements at 1 Hz 75 mA AC during formation of fouling layers by synthetic PS-PAA model particles in semi-dead-end filtration with the filtration setup described in Fig. S2b. Fouling layers were formed by semi-dead-end filtration at 1 bar TMP using 0.05 v/v% PS-PAA polystyrene-co-acrylic acid (PS-PAA) core–shell particles in 5 mM NaOH with a diameter of 1.4 µm synthesized with a method as described in Lorenzen et al.^25^. Particles were coagulated by addition of Ca^2+^ resulting in a decline in zeta potential from − 33.3 to − 3.2 mV (Malvern ZetaSizer Nano-ZS). The particles’ median diameter was 2.69 µm with d_10_ = 1.29 µm and d_90_ = 5.58. The* Ù*_3ω_ signal was collected at 75 mA AC with a frequency of 1 Hz. After analysis, the membrane was rinsed with DI water to remove and recover the fouling layer, which was quantified by gravimetric analysis after drying in an oven at 105 °C (Mettler AM100 analytical balance). Figure 3a shows how the flux declined during the 7300 s filtration, while *Ù*_3ω_ increased linearly with the total hydraulic resistance to filtration (Fig. 3b). Given that the semi-dead-end filtration of PS-PAA was carried out at constant pressure, the specific resistance to filtration can be assumed to be constant; that is, there is a direct proportionality between hydraulic resistance and thickness of fouling layer. This proportionality between amount of fouling and *Ù*_3ω_ is a result of the thermal conductivity of the fouling layer being lower than the surrounding feed suspension, which has a significantly higher water content.

The amount of fouling at the end of the filtration was assessed to be 208 g m^−2^ by gravimetric analysis, while the specific cake resistance was 3.52 × 10^12^ m kg^−1^. By assuming a particle density of 1050 kg m^−3^ and a porosity of cake of 0.45^25^, the average height of the cake was estimated to be 568 µm at the end of the filtration. The penetration depth of the signal was estimated to 105 µm in water at 1 Hz and 107 µm in polystyrene; hence, the penetration depth was within the estimated thickness of the cake layer. One explanation for the increasing signal and hydraulic resistance over time may be continuous buildup, but the compaction of the cake may also result in the signal increasing over time, the result of a higher proportion of particles with thermal conductivity lower than water.

### Sensing fouling during crossflow filtration

*Ù*_3ω_ signals were measured at 75 mA AC current and 1 Hz frequency through the wire on the membrane at a constant crossflow at 2.85 × 10^–2^ m s^−1^ and used as a reference experiment before the fouling experiments. After this, the membrane was fouled by filtration of milk (diluted from 3.5% fat content to 0.035% fat content in DI water) for 120 min in crossflow mode (2.85 × 10^–2^ m s^−1^), applying a TMP of 0.5 bar while measuring the *Ù*_3ω_ signal and permeate flux. After fouling, the membrane was cleaned by rinsing the membrane with a 0.5%w/w NaOH solution. After this, DI water was filtrated at a crossflow of 2.85 × 10^–2^ m s^−1^ and TMP = 0.5 bar to determine the reduction in hydraulic permeability after cleaning. The *Ù*_3ω_ signal was measured continuously during the filtration to determine the change in signal as a result of cleaning.

**Figure 4:**
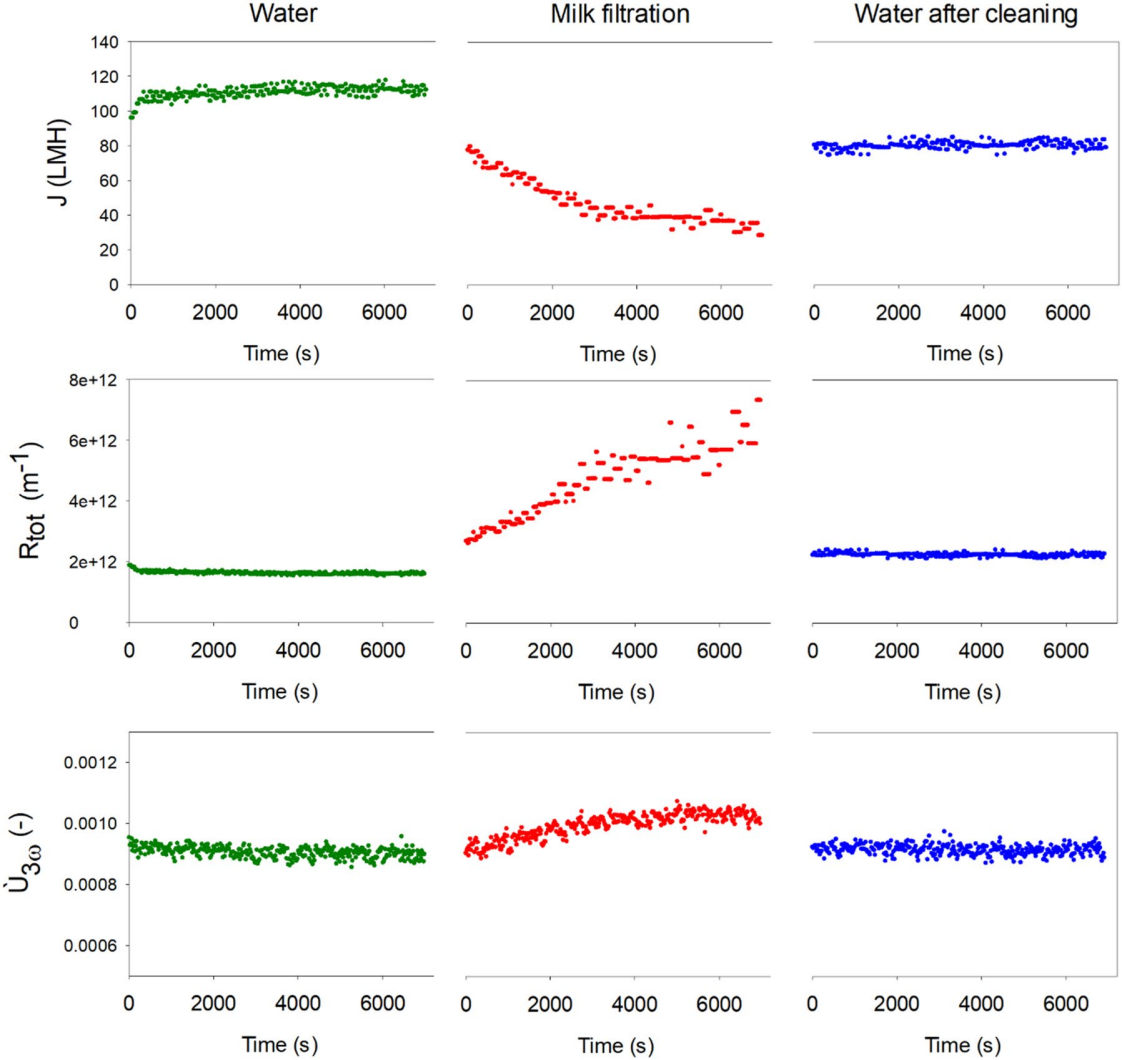
Development in flux (top) hydraulic resistance (middle) and Ù_3ω_ (bottom) over time during filtration of DI water (left), dilute milk (middle) and DI water after cleaning with NaOH (right).

The particle size distribution of the milk had a median diameter of 1.14 µm with *d*_10_ = 0.56 µm and *d*_90_ = 1.79 µm (measured by laser diffraction particle analyzer, LS 13320, Beckman Coulter), which resulted in high retention and fouling formation by the membrane with mean pore size of 60 nm. Figure 4 summarizes the results, showing a decline in permeate flux from 110 to 24 LMH during milk filtration as a result of fouling. *Ù*_3ω_ was constant at 9.1 × 10^–4^ ± 0.2 × 10^–4^ V during the initial filtration of DI water but increased continuously during fouling to reach a value of 10.2 × 10^–4^ ± 0.2 × 10^–5^ V during the last 5 min of filtration. This increase could be an effect of the change in thermal conductivity around the wire by the deposition of fat and casein, both of which have lower thermal conductivities than water, but it could also be an effect of the shielding towards heat convection by the crossflow, elevating the 3ω signal. After membrane cleaning, *Ù*_3ω_ was almost back to its original value; the measurement during the final DI water filtration was 9.14 × 10^–4^ ± 0.17 × 10^–4^ V. However, the permeate flux was only partly restored, with a flux of 80 LMH being reached, even though visual inspection of the membranes did not reveal residual fouling on the surface. This result and observation suggest that residual and irreversible fouling is present internally in the membrane, which may not affect the thermal conductivity of the membrane significantly enough to have an influence on the 3ω signal.

**Figure 5:**
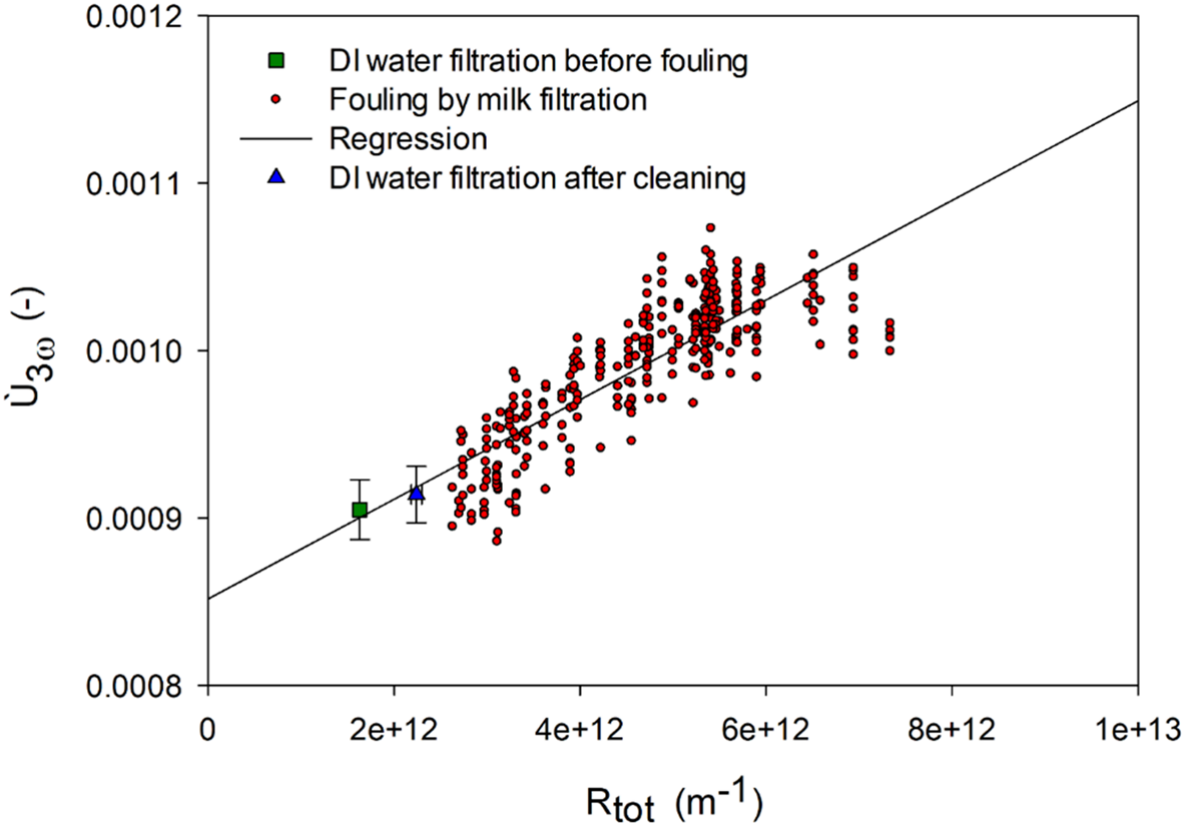
Ù_3ω_ plotted against hydraulic resistance to filtration from filtration of DI water, filtration of milk, and filtration of DI water after membrane cleaning. Plots of DI water filtrations are average values with standard deviations of the entire filtration.

In Fig. 5*Ù*_3ω_ is plotted against the hydraulic filtration resistance during filtration of DI water, fouling and filtration of DI water after cleaning. The results show a direct relationship between *Ù*_3ω_ signal and hydraulic resistance; that is, the more fouling, the higher the *Ù*_3ω_ signal. This relationship could again be a consequence of a thicker layer giving a lower thermal conductivity around the wire (thermal conductivity of milk powder is 0.07 W m^−1^ K^−1^^26^), or it could be a consequence of more shielding towards thermal convection by crossflow by thicker layers. At the time it was observed that the hydraulic resistance and *Ù*_3ω_ signal were not completely restored after cleaning, which may be an effect of irreversible fouling. However, a t-test showed that the measured hydraulic resistance after cleaning was significantly higher than before fouling, whereas *Ù*_3ω_ was not significantly different before and after filtration. This result may be a consequence of the formation of internal fouling in the membrane (e.g., pore blocking), which is irreversible and a greater contributor to the hydraulic resistance of the membrane while not significantly affecting the thermal conduction from the wire.

The milk filtration was repeated under the same conditions, which also led to an elevation in *Ù*_3ω_ as the membrane was fouled (Fig. S5a) and reduction after cleaning (Fig. S5b). However, the pressure was not kept constant. After 4350 s of filtration of dilute milk the TMP was released from 0.55 ± 0.01 bar to 0.35 ± 0.01 bar. This change resulted in a drop in hydraulic resistance, which can be explained by swelling of the fouling layer^27^. However, it also resulted in a gradual decline in *Ù*_3ω_, from 9.61 × 10^–4^ ± 0.12 × 10^–4^ to 9.36 × 10^–4^ ± 0.08 × 10^–4^. This decline can potentially be explained by the higher water content and higher thermal conductivity of the layer after swelling, a conjecture that should be investigated further in future studies.

## Discussion

By applying an AC current to 20 µm thick platinum wires integrated into ceramic ultrafiltration membranes, the (thermal conductivity dependent) *Ù*_3ω_ signal was measured by obtaining the amplitude of the third harmonic waveform, normalized with the applied voltage. This approach has the potential to serve as a real-time, non-destructive fouling sensor with the following features:

- *Ù*_3ω_ changes with the amount of fouling layer on the membrane surface as fouling is formed.
- *Ù*_3ω_ is reduced by crossflow and further reduced by increasing crossflow velocity, which is most prominent at low frequency AC.
- Fouling layer formation can be detected due to the difference in the layer’s thermal conductivity compared to suspension, but it can also be detected by protection against thermal convection by crossflow in crossflow filtration mode.
- The penetration depth of measuring *Ù*_3ω_ signals can be varied by the AC frequency. Thereby, at high frequencies, the *Ù*_3ω_ signal depends only on the thermal conductivity within the fouling layer, which potentially allows the layer to be characterized in terms of material or compactness, and so on. In contrast, AC frequency may be reduced to study how the fouling layer protects against heat convection by the surrounding environment (e.g., crossflow) to assess fouling layer thickness.

With these findings, the method has the potential to be used to monitor and reduce fouling formation, to optimize cleaning efficiency, and to distinguish between the loss of permeability due to an increasing amount of fouling and a loss due to changes in the composition of the fouling layer or mechanism. The current study was conducted with an ultrafiltration membrane, but the method is expected to also be applicable in microfiltration, nanofiltration, reverse osmosis, along with non-pressure driven membrane processes such as membrane distillation and forward osmosis. For other applications, the method has measured silica film thicknesses of 100 nm^28^, hence, it is plausible that the method can measure sub-micron fouling layers in e.g. reverse osmosis. For membrane distillation, the method may also be able to monitor temperature polarization.

The thermal conductivity measurements by 3ω sensing on a membrane can be expected to depend on different parameters at once, being, (1) crossflow velocity, (2) amount of fouling, (3) fouling material thermal conductivity, and (4) degree of compaction, which should be investigated in future studies. The effect of crossflow velocity on *Ù*_3ω_ signal should be determined by calibration measurements as a baseline before fouling experiments. It is expected that it will be possible to distinguish between effects of fouling quantity and type by varying the AC frequency, i.e. penetration depth. Over long-term filtration in e.g. a membrane bioreactor, *Ù*_3ω_ signal may initially increase due to formation of an insulating organic fouling layer, but if inorganic fouling would form over time, the *Ù*_3ω_ signal will decrease again as inorganic deposits have higher thermal conductivity. Furthermore, it should be investigated to what extend pore blocking can be monitored, as it must induce a significant change of the thermal conductivity of the membrane for the *Ù*_3ω_ signal to change.

In the current study, the 3ω method was shown to monitor fouling in a laboratory scale filtration module. For the method to be implemented into pilot or full scale, the membranes should be functionalized to be electrically conductive with a temperature coefficient of the electrical resistance to be able to measure *Ù*_3ω_ signals. For this, it may be more feasible to coat membranes with conductive material, or it may be possible to integrate platinum wires into the support layers or even in spacers in spiral wound modules. By making different parts of membranes electrically conductive, *Ù*_3ω_ signals can be measured locally to monitor the onset of fouling locally in modules before it develops, allowing for early stage, mild membrane cleaning.

## Methods

### Membrane sensor and filtration system

A ceramic flat sheet membrane Hybrid Technology Membrane manufactured by LiqTech Ceramics A/S with the dimensions 18 × 40 mm was applied. The membrane has a nominal pore size of 60 nm and the surface consists of a selective ZrO_2_ layer with a SiC support. Platinum wire (Goodfellow, PT00-WR-000110) with a diameter of 20 µm was fixed to the membrane surface with conducting glue (Loctite) on both ends. The thickness of the wire was verified by microscopy analysis as presented in Fig. S1. Crocodile clips were connected to aluminum foil to ensure electrical contact with the platinum wire. An AC current source (Keithley 6221) was applied to generate the 3ω signals. It was connected to the aluminum foils along with a 16 bit 250 kS/s data acquisition card (National Instruments USB-6210). MATLAB scripts were developed to collect measured voltage over time from the data acquisition card. The sampling rate was 2 kHz. For continuous measurements during filtration, the signal was found by dividing 100 s measurements into five equal sized arrays and performing Fourier transformations on each of them; that is, 350 *U*_3ω_ signals were measured throughout a longer continuous run with 70 successive measurements. Outliers, defined as *U*_3ω_ signals more than three standard deviations away from the median in moving intervals of 50 data points, were discarded.

The filtration cell had a feed inlet, a permeate outlet and a retentate outlet (Fig. 1b). Additionally, the permeate and retentate lines were equipped with valves that can be opened or closed to allow for permeation and crossflow. For crossflow filtration, a membrane pump with variable pump speed (Vetus, type WP240B) drew suspension from a beaker to the membrane cell (Fig. S2a). A pressure meter (Honeywell, 2118610131) was connected on the feed and retentate lines and a manual flow meter (MCC) was connected to the retentate outlet before a needle valve to adjust flow and pressure together with the pump speed. The retentate was redirected to the feed beaker while the permeate was collected in a beaker placed on a scale (Kern, PCB 6000-1). The mass and pressure data were collected every ten seconds using MATLAB.

For semi-dead-end filtrations, a steel container was filled with feed suspension and pressurized with compressed air (Fig. S2b). The container was connected to the feed inlet in the membrane cell, and the retentate valve was closed for semi-dead-end operation. The permeate was collected in a beaker placed on a scale (Kern, PCB 6000-1) and weight was logged every five seconds using a MATLAB script.

### Signal analysis

The recorded signals were sinusoidal curves of voltage over time. As a direct demonstration of the 3ω signal, a sinusoidal function with frequency ω was fitted to the measured voltage (Fig. S6a). By finding the residual between the fit and the measured data, a sinusoidal curve with a frequency of 3 times the AC frequency appears, the amplitude of which is the 3ω signal, *U*_3ω_^23^. This value could also be found by Fourier transformation of the measured voltage (Fig. S6b). For short measurements, data were divided into five fractions of 10 periods each, and *U*_3ω_ was found for each by Fourier analysis.

The *U*_3ω_ signal depends on the magnitude of the temperature oscillation in the Pt wire (*ΔT*_AC_) that are induced by the oscillating AC current, and the measured voltage over the platinum wire, *U*_1ω_, as follows^23,29^:

1 $${U}_{3\omega }=\frac{1}{2}{U}_{1\omega }\beta \Delta {T}_{AC},$$

where β is the temperature coefficient of resistance of the Pt wire. Under the assumption that the width of the boundary layer is lower than the thermal wavelength (i.e., the penetration depth), the magnitude of temperature oscillations can be described as:

2 $$\Delta {T}_{AC}=\frac{P}{l\pi \kappa }\left(-\frac{1}{2}\mathrm{ln}\left(\omega \right)+k\right),$$

in which *P* is the Joule heating, l is the length of the wire, *κ* is the thermal conductivity of materials around the wire, *ω* is the angular frequency and *k* is a complex geometric constant^23,29^. Equations (1) and (2) can be combined to express *U*_3ω_^23,29^ as follows:

3 $${U}_{3\omega }=\frac{1}{2}{U}_{1\omega }\beta \frac{P}{l\pi \kappa }\left(-\frac{1}{2}\mathrm{ln}\left(\omega \right)+k\right)$$

The U_3ω_ signal is inversely proportional to the thermal conductivity, and decreases with AC frequency. It also increases with the current, due to higher Joule heating, which also affects the measured *U*_0_. The electrical resistance of the platinum wire increases with temperature, meaning that the measured voltage at a given current will increase with temperature. This increase will also lead to an increase in *U*_3ω_. To compensate, the *U*_3ω_ signal is normalized with respect to *U*_1ω_ as follows:

4 $${\stackrel{`}{U}}_{3\omega }=\frac{{U}_{3\omega }}{{U}_{1\omega }},$$

Measurements of *U*_1ω_ and *U*_3ω_ at varying temperatures confirm that the *Ù*_3ω_, at AC frequencies 0.1, 1 and 10 Hz, are independent of temperature (Fig. S3). In addition, measurements of the *Ù*_3ω_ signal in DI water show that the signal is stable over two hours of measurements at 75 mA 1 Hz AC frequency.

The thermal wavelength, that is, penetration depth, depends on the AC frequency and the thermal diffusivity, *D*, as follows:

5 $$\lambda =\sqrt{\frac{D}{2\omega }}$$

Hence, the penetration depth can be expanded by reducing AC frequency.

### Measurements of 3ω signals in different environments and fouling conditions

The experiments aimed to investigate the capability of the 3ω method to detect variations in flow and deposits on the membrane. Except for continuous measurements during fouling, measurements of *Ù*_3ω_ were repeated three times at 0.1, 1 and 10 Hz AC frequencies and one time for 0.01 Hz AC frequencies, with five waveforms of 10 periods collected for 1 and 10 Hz AC frequencies and only one waveform was collected at 0.01 Hz AC frequency. In addition, continuous measurements, which were done to follow fouling formation during filtration, were conducted as 70 repetitions of 100 s measurements. As a reference for interpretation of changes in *Ù*_3ω_ over time, it was demonstrated that the signal does not change over time while applying a 1 Hz AC current of 75 mA for 2 h (Fig. S7). This normalized standard deviation of the measured signal is 0.8%, which corresponds to a signal to noise ratio of 120. Measured *U*_3ω_ more than three standard deviations away from the median in moving intervals of 50 data points were defined as outliers in the continuous measurements.

### Analysis of filtration data

Permeate flux (*J*) was calculated from the increase in mass of collected permeate over time. From this value, the total hydraulic resistance, which is the sum of the membrane and fouling layer resistances, was calculated as follows:

6 $${R}_{tot}={R}_{m}+{R}_{f}=\frac{TMP}{J\mu },$$

where µ is the dynamic viscosity of water. Assuming cake formation is the mechanism of fouling, the fouling layer resistance can be described in terms of the cake resistance as follows:

7 $${R}_{f}={R}_{c}=\alpha \times {m}_{c},$$

in which *α* is the specific cake resistance (m kg^−1^) and *m*_c_ is the specific amount of mass (kg m^−2^).

For dead-end and semi-dead-end filtration, *m*_c_ can be assumed to increase linearly with the volume of permeate, *V*_p_, which forms the basis of the following equation:

8 $$\frac{t}{{V}_{p}}=\alpha \frac{\mu C}{2{A}^{2}TMP}{V}_{p}+{R}_{m}\frac{\mu }{A\times TMP}$$

A is the membrane area (m^2^) and C is the concentration of suspended particles retained by the membrane (kg m^−3^). It follows that plotting *t*/*V*_p_ against *V*_p_ (Ruth plot) will give a straight line in the region with cake formation as the dominating fouling mechanism. From the slope, the specific cake resistance, *α* (m kg^−1^)*,* is determined.

### Supplementary Information

Supplementary Figures.

## Data Availability

The datasets used and/or analyzed during the current study are available from the corresponding author on reasonable request.
