# Supplementary material for: Membrane fouling monitoring by 3ω sensing
Source: Sci Rep. 2023 Sep 14;13:15237. doi: 10.1038/s41598-023-42337-1 (PMC10502093; doi:10.1038/s41598-023-42337-1)
Supplement: Supplementary file 1 — Supplementary Figures. [file 41598_2023_42337_MOESM1_ESM.docx]

**Supplementary information**

**Membrane fouling monitoring by 3ω sensing**

Mads Koustrup Jørgensen^1,*^, Frederikke Kildeberg Paulsen^1^, Anders Bentien^2^, Astrid Ræbild Kjul^1^, Maiken Poulsen^1^, Louise Mailund Mikkelsen^1^, Nikitha Thavaneswaran^1^, Simon Abildgaard Hansen^1^, Pernille Krogsager Jensen^1^, Jacob Andersen^1^, David N. Østedgaard-Munck^3^, Jan Dimon Bendtsen^4^, Morten Lykkegaard Christensen^1^

^1^Center for Membrane Technology, Department of Chemistry and Bioscience, Aalborg University, DK-9220 Aalborg East, Denmark

^2^Department of Biological and Chemical Engineering, Aarhus University, Åbogade 40, DK-8200 Aarhus N, Denmark

^3^LiqTech International A/S, Benshøj Industrivej 24, DK-9500 Hobro, Denmark

^4^Department of Electronic Systems, Aalborg University, Fredrik Bajers Vej 7, DK-9220 Aalborg Øst

*Correspondence: [mkj@bio.aau.dk](mailto:mkj@bio.aau.dk)

**This file includes:**

Supplementary Figures S1-S7.


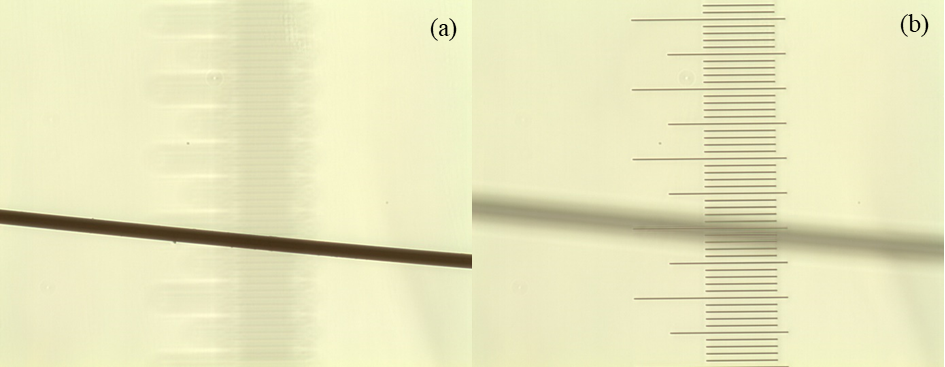


Figure S1: Microscopy image of platinum wire with wire (a) and scale (b) in focus. Every bar on the scale is 10 μm. The microscope is an Axioskop epifluorescence microscope (Carl Zeiss, Oberkochen, Germany, 1000x magnification).


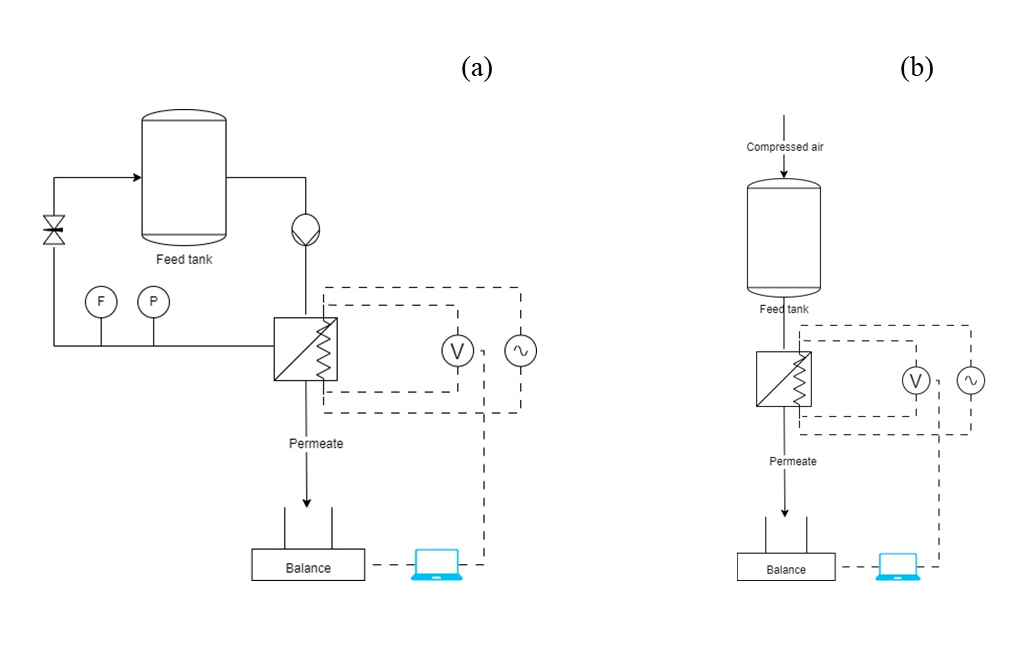


Figure S2: Schematic of crossflow filtration setup (a) and semi-dead-end filtration setup (b).

Figure S3: Ù_3ω_ collected at 75 mA AC current through a platinum wire on a membrane in DI water collected at 0.1, 1 and 10 Hz.

Figure S4: Ù_3ω_ collected at 75 mA AC current with a frequency of 1 Hz and a crossflow velocity of 0.043 m s^-1^ for varying permeate fluxes during filtration of DI water. Data are average values of three replicates.

Figure S5: Ù_3ω_ and TMP and hydraulic resistance to filtration plotted against time during filtration of dilute milk samples (0.035% fat content) (a), and Ù_3ω_ plotted against hydraulic resistance during filtration of DI water, dilute milk and DI water after membrane cleaning (b). TMP was released from 0.55 ±0.01 bar to 0.35 ±0.01 bar after 4350 s of filtration.


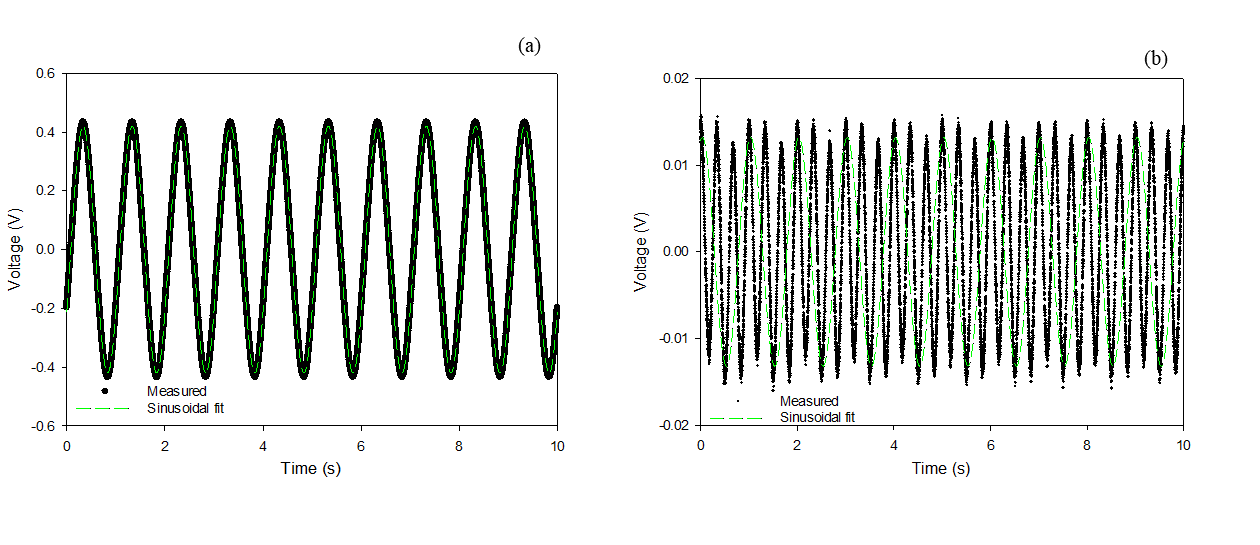


Figure S6: Measured voltage through platinum wire on membrane in air with 75 mA current at 1 Hz AC frequency along with sinusoidal fit (a) and residual between fit and measured voltage, again with a sinusoidal fit to the residual with an oscillation of 3 Hz and amplitude of U_3ω_ (b).

Figure S7: Ù_3ω_ collected at 75 mA AC current with a frequency of 1 Hz in water at room temperature as a reference for continuous filtration experiments.
